# LYPD8 regulates the proliferation and migration of colorectal cancer cells through inhibiting the secretion of IL-6 and TNF-α

**DOI:** 10.3892/or.2021.8004

**Published:** 2021-03-05

**Authors:** Jianbin Xu, Jun Qian, Wei Zhang, Engeng Chen, Guolin Zhang, Gaoyang Cao, Fei Wang, Xuning Shen, Wei Zhou, Zhangfa Song

Oncol Rep 41: 2389-2395, 2019; DOI: 10.3892/or.2019.7034

Following the publication of the above paper, the authors realized that, in their follow-up experiments, the STAT3 and p65 antibodies they used had already expired prior to the results being published. This affected the confidence that the authors could place in the results published in Figs. 1 and 3E. In addition, the findings were also inconsistent with Fig. 4A and B, potentially causing confusion for the readers.

The authors therefore repeated some of these experiments with newly purchased antibodies; they also changed the RT-qPCR results. In the published manuscript, the authors investigated the expression of LYPD8 mRNA expression in tissues from stages I, II, and III whereas in the revised manuscript they have investigated stages II and IV. In addition, the authors have supplemented the manuscript with new transwell assays. The revised figures are presented on the next two pages (Figs. 1–4). Repeating these particular experiments has resulted in the following changes being necessary to the text of the published paper (changes are highlighted in bold):

i) The fourth sentence in the Abstract, on p. 2389, should read as follows: “The results revealed that the expression of LYPD8 was significantly reduced in the CRC tissue compared with that in precancerous tissue and normal tissue, particularly in stage **IV** tissue.” (‘III’ has been changed to ‘IV’).

ii) In the Materials and methods section, “*Histological analysis*” subsection on p. 2390, the last three sentences should be replaced with the following text: “**The histological sections were then stained with the DAB Kit (cat. no. PV-9000; ZSGB-BIO, Beijing, China). All sections were observed under a bright-field microscope (Nikon Corporation, Tokyo, Japan).**”

iii) In the “*Cell culture*” subsection in the right-hand column, the first four sentences should be revised to the following: “**Four** CRC cell lines **(SW480, SW620, HCT116 and RKO)** were used. **SW480 (ATCC^®^ CCL-228™, organism, human; tissue, colon; disease, colorectal adenocarcinoma), SW620 (ATCC^®^ CCL-227™, organism, human; tissue, colon; derived from metastatic site, lymph node; disease, colorectal adenocarcinoma), HCT116 (ATCC^®^ CCL-247™, organism, human; tissue, colon; disease, colorectal carcinoma) and RKO (ATCC^®^ CRL-2577™, organism, human; tissue, colon; disease, carcinoma) cells were purchased from the American Type Culture Collection (Manassas, VA, USA). The four** cell lines within passages 10 were used in all experiments, **and the cell lines** were maintained at 37°C in a humidified incubator containing 5% CO_2_”. Also, in line 8 of p. 2391, the cell lines here should be changed to “SW480, **SW620 and HCT116** cells”, and on line 11, “HT29 cells” should be changed to “**RKO cells**”.

iv) In the Results section, the following changes to the text are necessary: In the “*Correlation of the expression of LYPD8 with STAT3/P65 phosphorylation and IL-6/TNF-α secretion in patients with CRC*” subsection, in the second sentence, “immunofluorescence” should have been written as “**immunohistochemistry**”, and the fourth sentence should have read as follows: “The results of the western blotting showed that the levels of p-P65/P65 and p-STAT3/STAT3 gradually increased between stage **II and IV** (Fig. 1B and C).” Then, the three sentences starting on line 7 on p. 2391 should now read as follows: “Following this, the gene expression levels of LYWPD8 in stage **II and IV** CRC tissue, precancerous tissue, and normal tissue were assessed using RT-qPCR analysis (Fig. 2C). Compared with the precancerous tissue and normal tissue, the gene expression of LYPD8 was significantly reduced in stage **II and IV** tissues. Furthermore, the expression of LYPD8 was reduced in stage **IV** tissue compared with that in stage **II** tissue.”

v) In the subsequent subsection, “*Construction and overexpression of LYPD8 in CRC cells*”, the first sentence should have read as follows: “The plasmid DNA for overexpressing LYPD8 was constructed using the eukaryotic expression vector (pIRES2), as shown in Fig. 3A and B, and the relative expression levels of LYPD8 in the **RTO**, SW480 **HCT116** and SW620 cells were examined by RT-qPCR analysis.

vi) In the “*Overexpression of LYPD8 inhibits CRC cell proliferation and migration*” subsection, the penultimate sentence as it appears towards the foot of p. 2392 should now read as follows: “As shown in Fig. 4C and D, a more marked inhibitory effect on cell migration was observed in the LYPD8 OE group compared with that in the **control, LYPD8 OE + IL-6 and LYPD8 OE + TNF-α groups.**”

vi) In the Discussion, the sentence starting on p. 2394, right-hand column, line 10 should read as follows: “By contrast, the expression of LYPD8 was significantly reduced in stage **II and IV** CRC tissues.”

vii) Finally, some revisions were necessary to the descriptions in the figure legends for Figs. 1, 2 and 4, as follows (only the affected text is included, and the changes are indicated in bold):

Figure 1. STAT3 and P65 are activated in colonic tumor tissues from patients. (A) Representative **immunohistochemistry** images revealing activated STAT3 and P65 in colonic cancer tissue and precancerous tissue. Scale bar, 100 µm. (B) Representative western blotting revealing the expression of p-P65, P65, p-STAT3 and STAT3 in stages **II and IV** colonic tumor tissues. GAPDH was used as a control. (C) Band intensities of western blotting for p-P65/P65 and p-STAT3/STAT3 in stage **II and IV** tissues were analyzed. The data are reported as the mean ± standard deviation of experiments (n=4). **P<0.05, phosphorylation levels of STAT3 in **stage II** tissues vs. in **stage IV** tissues.

Figure 2. Association between IL-6/TNF-α and the expression of LYPD8 in colonic tumor tissue, precancerous tissue and normal tissue at different stages. (A) IL-6 and (B) TNF-α secretion were analyzed by ELISA in stage II and IV colonic tumor tissue and precancerous tissue. (C) Gene expression of LYPD8 in stage **II and IV** colonic tumor tissue and precancerous tissue. β-actin was used as a control. The data are reported as the mean ± standard deviation of experiments (n=6). **P<0.01, LYPD8 mRNA expression of normal tissue, precancerous tissue vs. colonic tumor tissue in stage **II and IV** tissues.

Figure 4. Effects of the overexpression of LYPD8 on SW480 cell proliferation and migration. (A) Cell viability of the Control, LYPD, **LYPD8 OE + IL-6 and LYPD8 OE + TNF-α** groups of SW480 cells. (B) SW480 cells were treated with different concentrations (0.5, 1 and 2 µM) of niclosamide and different concentrations (5, 15 and 30 µM) of JSH-23, respectively. (C) Numbers of migratory SW480 cells from the **Control, LYPD8, LYPD8 OE + IL-6 and LYPD8 OE + TNF-α** groups. (D) Transwell assay of SW480 cells from the (a) **Control, (b) LYPD8 OE, (c) LYPD8 OE + IL-6 and (d) LYPD8 OE + TNF-α** groups (magnification, ×200).

Note that the replacement of the original figures and these revisions made to the text do not drastically alter the overall conclusions reported in the study. The authors are very grateful to the Editor of *Oncology Reports* for allowing them the opportunity to publish this Corrigendum; furthermore, they apologize for any inconvenience caused to the readership of the Journal.

## Figures and Tables

**Figure 1.**
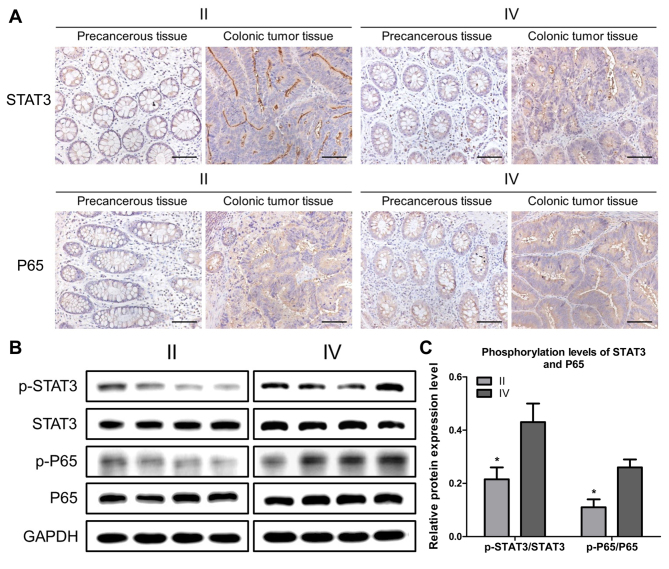
STAT3 and P65 are activated in colonic tumor tissues from patients. (A) Representative **immunohistochemistry** images revealing activated STAT3 and P65 in colonic cancer tissue and precancerous tissue. Scale bar, 100 µm. (B) Representative western blotting revealing the expression of p-P65, P65, p-STAT3 and STAT3 in stages **II and IV** colonic tumor tissues. GAPDH was used as a control. (C) Band intensities of western blotting for p-P65/P65 and p-STAT3/STAT3 in stage **II and IV** tissues were analyzed. The data are reported as the mean ± standard deviation of experiments (n=4). **P<0.05, phosphorylation levels of STAT3 in **stage II** tissues vs. in **stage IV** tissues. STAT3, signal transducer and activator of transcription 3; p-, phosphorylated.

**Figure 2.**
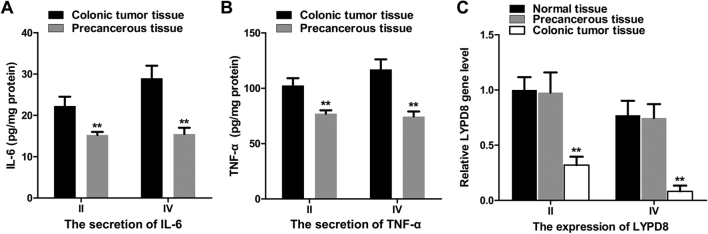
Association between IL-6/TNF-α and the expression of LYPD8 in colonic tumor tissue, precancerous tissue and normal tissue at different stages. (A) IL-6 and (B) TNF-α secretion were analyzed by ELISA in stage II and IV colonic tumor tissue and precancerous tissue. (C) Gene expression of LYPD8 in stage **II and IV** colonic tumor tissue and precancerous tissue. β-actin was used as a control. The data are reported as the mean ± standard deviation of experiments (n=6). **P<0.01, LYPD8 mRNA expression of normal tissue, precancerous tissue vs. colonic tumor tissue in stage **II and IV** tissues. LYPD8, Ly6/Plaur domain-containing 8; IL-6, interleukin-6; TNF-α, tumor necrosis factor-α.

**Figure 3.**
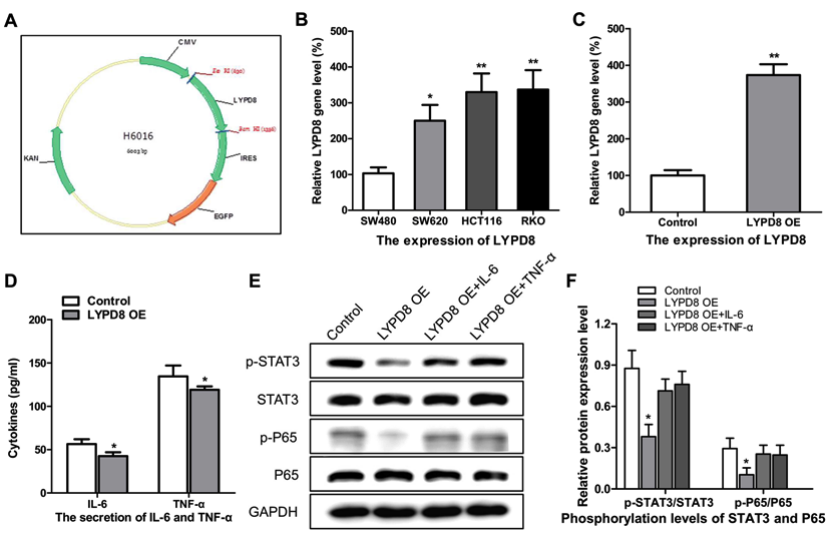
Effects of the overexpression of LYPD8 on IL-6/TNF-α secretion and STAT3/P65 dephosphorylation in CRC cells. (A) cDNA coding for the LYPD8 gene was cloned into the eukaryotic expression vector. (B) Expression levels of LYPD8 in different CRC cells were determined by RT-qPCR analysis, using β-actin as a control. (C) Overexpression of LYPD8 via transient transfection into SW480 cells was confirmed by RT-qPCR analysis. (D) IL-6/TNF-α secretion was analyzed by ELISA in LYPD8 OE groups of SW480 cells. (E) Western blotting revealed expression levels of p-P65, P65, p-STAT3 and STAT3 in the control, LYPD8 OE, LYPD8 OE + TNF-α and LYPD8 OE + IL-6 groups of SW480 cells. GAPDH was used as a control. (F) Band intensities of western blotting for p-P65/P65 and p-STAT3/STAT3 in the control, LYPD8 OE, LYPD8 OE + TNF-α and LYPD8 OE + IL-6 groups were analyzed. The data are reported as the mean ± standard deviation of experiments (n=4). *P<0.05, phosphorylation levels of P65 and STAT3 in LYPD8 OE groups vs. control, LYPD8 OE + TNF-α and LYPD8 OE + IL-6 groups; **P<0.01, expression of LYPD8 in control group vs. LYPD8 OE group. Control, empty pIRES2; LYPD8, Ly6/Plaur domain-containing 8; OE, overexpression; IL-6, interleukin-6; TNF-α, tumor necrosis factor-α; STAT3, signal transducer and activator of transcription 3; p-, phosphorylated; RT-qPCR, reverse transcription-quantitative polymerase chain reaction.

**Figure 4.**
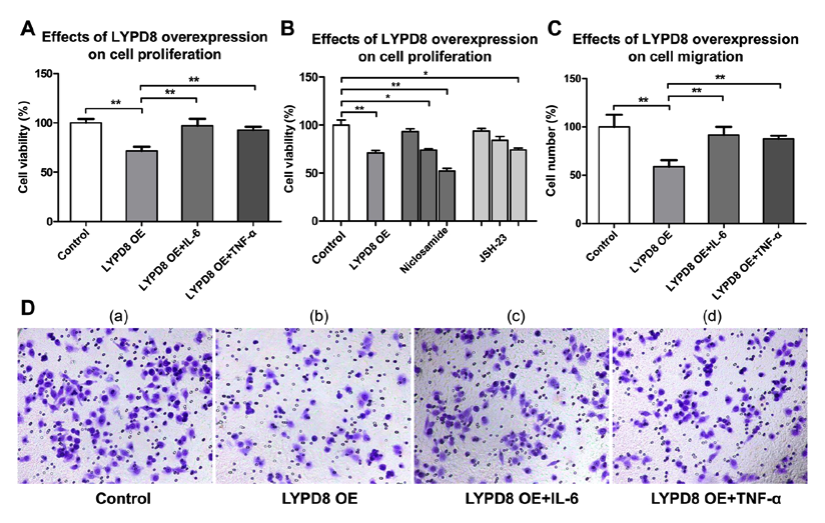
Effects of the overexpression of LYPD8 on SW480 cell proliferation and migration. (A) Cell viability of the Control, LYPD, **LYPD8 OE + IL-6 and LYPD8 OE + TNF-α** groups of SW480 cells. (B) SW480 cells were treated with different concentrations (0.5, 1 and 2 µM) of niclosamide and different concentrations (5, 15 and 30 µM) of JSH-23, respectively. (C) Numbers of migratory SW480 cells from the **Control, LYPD8, LYPD8 OE + IL-6 and LYPD8 OE + TNF-α** groups. (D) Transwell assay of SW480 cells from the (a) **Control, (b) LYPD8 OE, (c) LYPD8 OE + IL-6 and (d) LYPD8 OE + TNF-α** groups (magnification, ×200). The data are reported as the mean ± standard deviation of experiments (n=4). *P<0.05, **P<0.01. Control, empty pIRES2 group; LYPD8, Ly6/Plaur domain-containing 8; OE, overexpression; IL-6, interleukin-6; TNF-α, tumor necrosis factor-α.

